# Reduce or not reduce: a practical guide to calculate sample size for various experimental design in studies involving animals

**DOI:** 10.3389/fphar.2026.1786234

**Published:** 2026-05-28

**Authors:** Jean Xavier Mazoit, Patrick Gonin

**Affiliations:** 1 Animal Ethics Committee CEEA26 (Comité d’Ethique en Expérimentale Animale 26), Université Paris-Saclay, Villejuif, France; 2 Ex. Laboratoire d’anesthésie, Université Paris-Saclay and INSERM U1195, Villejuif, France; 3 Université Paris-Saclay, Gustave Roussy, Inserm, Unité Analyse Moléculaire et Imagerie de la Maladie Cancéreuse, Plateforme d’Evaluation Préclinique, Comité d'Ethique n°26, Villejuif, France

**Keywords:** animal experimentation, experimentaldesign, pharmacokinetics and pharmacodynamics, preclinicalresearch, reduction principle (3Rs), sample size calculation, statistical power

## Abstract

The ethical imperative to reduce the use of animals in scientific experimentation has become increasingly prominent, driven by societal expectations, regulatory frameworks, and concerns regarding the reproducibility of preclinical research. While the principle of Reduction, one of the 3Rs originally defined by Russell and Burch, is widely endorsed, its practical implementation remains challenging. In particular, inappropriate experimental designs, inadequate statistical planning, and insufficient reporting standards continue to undermine both scientific validity and ethical justification, often leading either to the unnecessary use of animals or to underpowered studies whose results lack reliability. This review provides an updated and practical framework for determining the appropriate number of animals required in experimental biology and pharmacology. Building on earlier methodological recommendations, it emphasizes the central role of rigorous study planning, including the clear definition of objectives and primary endpoints, appropriate experimental design, randomization, blinding, and the judicious use of control groups. Special attention is given to factorial designs and the inclusion of interactions, which often allow substantial reductions in animal numbers without compromising statistical power. The review also discusses conditional procedures, pilot studies, interim analyses, and the frequent confusion between biological replicates, technical replicates, and true experimental replication. The statistical foundations of *a priori* sample size calculation are revisited, including the concepts of Type I and Type II errors, effect size, variability, and corrections for multiple comparisons. Practical guidance is provided for common experimental contexts, including comparisons of continuous and categorical outcomes, analysis of variance, survival analysis, pharmacokinetic and pharmacodynamic studies, and dose–response experiments. Emerging approaches, such as sequential and Bayesian methods, are also discussed with respect to their potential for reduction and their limitations. Finally, this review highlights the ethical and scientific risks associated with excessive reduction leading to underpowered studies. It argues that optimal reduction does not mean using as few animals as possible, but rather using the right number, justified by sound experimental design and statistical reasoning. By promoting transparent planning and robust methodology, this work aims to support both ethical responsibility and scientific rigor in animal-based research.

## 1 Introduction

Growing societal expectations and ethical concerns increasingly drive efforts to reduce the use of animals in scientific experimentation (Abarkan et al., 2022). As early as 1959, William Russell and Rex Burch laid the ethical foundations for animal experimentation by defining the concept of the 3Rs (Russell and Burch, 1992). The second R (reduce) is part of this attempt to eventually limit the use of animals, with the final aim of eliminating it altogether in European countries (EU, 2010). Unfortunately, most studies in the literature do not report how the authors calculated the number of animals needed, whether the study was randomized, or whether the observations were blinded (Landis et al., 2012). Statistics are notoriously wrong in biological journals including the major ones (Ioannidis, 2006; Hayden, 2013; Nuzzo, 2014). While medical and psychological journals have regularly increased their standards in the past 50–60 years, a large number of biological journals have maintained the same low level of statistical analysis. Most medical journals have consultant statisticians who are systematically or on-demand involved in the reviewing process. Conversely, most biologists have a low background in statistics and applied mathematics, and often consider statistics negligible if not simply annoying. The consequence is that, in a great number of biology journals, the number of animals studied seems to be mostly “pulled out of the air.” This attitude causes major mistakes in articles that may induce erroneous scientific results and ethical issues.

What are the bases for calculating the number of subjects needed? The first thing to realize is that this is a bet on the future. In classical inferential statistics, there are two approaches: Ronald Fisher’s (*a posteriori* approach), which consists of looking only at the raw probability once the experiment is done, and Neyman-Pearson approach, which considers alpha and beta risks, and does so *a priori* (Lehmann, 1993; Perezgonzalez, 2015). It is this last principle that allows the number of subjects required to be calculated *a priori*. Currently, we are returning in part to Fisher’s approach for the presentation of results (Wasserstein and Lazar, 2019), but the calculation of the number of animals required is based on the second approach.

This review is an update of the recommendations made by Michael Festing and Douglas Altman more than 20 years ago (Festing and Altman, 2002), with particular attention to the second R (Reduce). We will discuss study planning, ways to calculate the minimum number of animals needed for a study, the danger of trying to reduce too much, and finally, how to calculate the right number of animals needed. We will not address the problems not directly related to statistical analysis, but which can lead to inflation in the number of animals through a side effect, such as the choice of sex or the management of breeding. A practical addendum will list a choice of computer resources available for performing the calculations.

### 1.1 Glossary and definitions

The glossary can be found in Box 1.

BOX 1 **Definitions of terms used in statistics.**

Error. In statistics, an error (or measurement error) is not a mistake but the extent of variability observed from one sample to another.

Population. In statistics, a population is a set on which observations are made.

Procedure. An experimental procedure corresponds to a sub-question of an overall scientific project.

Project. Here, we mean a scientific study that aims to answer a scientific question.

Replicate or repeat. A repeat is the act of creating a sample. Subjects are repeatedly drawn from a population to create a representative sample of that population.

Replication. Replication consists of repeating the entire experiment.

Sample. In statistics, a sample is a subset of a population drawn at random, like drawing colored balls from an urn.

Treatment. In statistics, a treatment is a condition applied to a statistical population. It is not necessarily a molecule.

Type I error. This is the risk of finding a difference when there is none (false positive). The type I error has a probability (alpha) usually set at 5% or 1% (0.05 or 0.01). Please note that in most cases, the alpha risk must be two-tailed. Experimenters often make the mistake of keeping the default value in the software, which may be that of a one-tailed test. The use of a one-tailed test must be fully justified.

Type II error. This is the risk of not finding a difference when one exists (false negative). The type II error has a probability (beta). The power is equal to 1 – beta. When calculating power, 80% is often chosen, more rarely 90% when the cost of a false negative is particularly high. The higher the power chosen, the greater the number of subjects required. Here too, care must be taken with the default values in the software.

Variability. This refers to the variability of the criterion used to calculate the number of subjects required. In general, for continuous variables, this is the standard deviation of the control population (SD), often expressed as a relative value in relation to the mean. This variability is data that comes either from the literature or from previous laboratory data. The greater the variability, the greater the number of subjects required.

Effect size. This is the magnitude of the expected effect between two statistical populations (e.g., placebo and active treatment). Jacob Cohen defined the effect size for all kinds of comparisons (Cohen, 1988). For example, Cohen’s d refers to the difference between means. It is the ratio between the difference in effect and variability (m1 – m2)/SD^1^. The smaller the expected difference, the greater the number of subjects needed to show this difference, and *vice versa*. Cohen’s d applies to Student’s t-test. Traditionally, Cohen’s d values of 0.2, 0.5, and 0.8 are considered small, medium, and large, respectively. There are other kinds of effect sizes, such as Cohen’s f, which applies to the Fisher-Snedecor test, Cohen’s omega (ω), which applies to the Chi2 test, but also the coefficient of determination (R^2^), the Odds Ratio, the number of subjects to be treated (NNT), etc.

## 2 The ethics and logic of sample size reduction

### 2.1 Study objective(s) and primary endpoint

At the project design stage, the following elements must be clearly defined: 1) the objective(s) of the study, 2) the primary endpoint and any secondary endpoints. The number of subjects required (usually referred to as the power analysis) will be based on the primary endpoint. It is common practice—and recommended—to have one endpoint per procedure if the project involves several distinct procedures.

After selecting and presenting the objectives and endpoints, it is necessary to plan the blinding and randomization techniques (Landis et al., 2012).

Thus, after defining the endpoint(s), the first step will be to develop one or more experimental designs.

### 2.2 Design of experiments

An experimental design is a study protocol that allows the study of a response as a function of variables (or factors) (Armitage and Berry, 1994; Festing and Altman, 2002). An experimental design allows for the control of factors that may influence the results. For example, sex, age, cage location in the rack/room, day of the week, etc. Adequate designs allow keeping the number of subjects to be included to a minimum. If the authors of the project plan to analyze their results using ANOVA, it is essential that they have considered the design of the experiment beforehand, as ANOVA is an experimental design by itself. Complete factorial designs that include interactions are the most commonly used and are generally recommended. They are moderately economical in terms of the number of animals required; however, more complex designs are difficult to implement in biological research. They can be improved by stratification (e.g., by sex or age, etc). This should not lead to an indiscriminate increase in the number of animals, inasmuch as sex, for example, is not considered as a discriminating factor in most experiments.

At the project design step, researchers very often present a one-way ANOVA, even though there are 2, 3, 4, etc., factors. A simple example is a study of two types of mice, A and B (e.g., wild and KO), and two treatments, 1 and 2 (e.g., placebo and anti-cancer). There are four groups: A1, A2, B1, and B2. This is a complete factorial design that will be analyzed *a posteriori* by a two-way factorial ANOVA with interactions, rather than by a one-way ANOVA with four independent groups (see paragraph 4.4 below). This type of design allows considering in advance which post-hoc comparisons will be relevant (see below, the section on multiple comparisons, paragraph 3.2).

A second consideration that must be considered along with the experimental design is the inclusion of conditional procedures (known as Go-NoGo or if, then, else).

### 2.3 Conditional procedures and pilot studies

These two types of procedures may be recommended under certain conditions.

The outcome of one procedure may determine whether other procedures are carried out in a sequence often referred to as Go/No go, or if, then, else. This solution needs to be favored. It may save many animals, because some procedures are only carried out under certain conditions. The same applies to proof-of-concept or pilot procedures when they are well understood. Indeed, they do not require statistical analysis and are most often carried out with a very small number of animals. They lead either to the abandonment of the project or to a reduction in the number of subjects studied in subsequent procedures. A common example is the search for a dose or measurement time as the initial procedure in a more general project.

### 2.4 Control groups

Researchers often add unnecessary groups: negative control, positive control, etc. This practice originates from molecular or cell biology (particularly cell culture). It is important to note that in this case, these groups are used to verify the validity of the experiment, not to be included in a statistical analysis. Control groups are useful only if they will be incorporated in the final statistical analysis. Clearly, positive controls are no more useful in animal experimentation than in human trials (except in very rare cases where the focus is on reducing toxicity). Negative controls (often containing the solvent of the molecule(s) being tested) are only useful if there is no active comparator, as in human trials.

### 2.5 Should repetitions be accepted?

**FIGURE 1:**
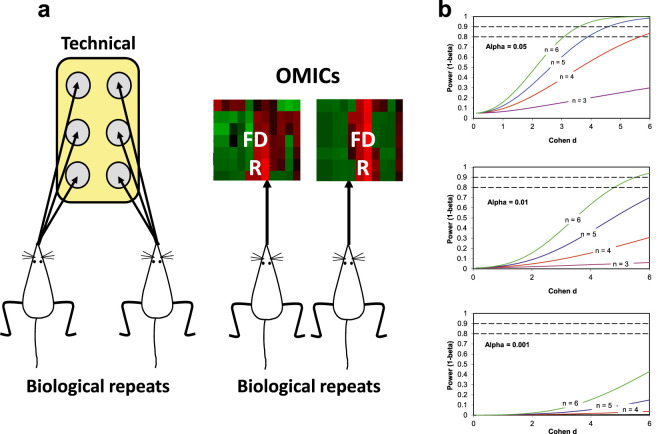
**(a)** Differences between biological and technical repeats. Left: the three wells in the plate represent three technical repeats from the same mouse allowing to decrease the measurement error by averaging the three values and possibly eliminate outliers. Statistical inference cannot be made on technical repeats, but on biological repeats (mice). Right: each heatmap comes from a different mouse, then inference is done on the unique value from each mouse. Please note that a previous analysis has be done on each map by using false discovery rate (Benjamini and Hochberg, 1995). Therefore, the calculation of the number of animals needed should be done as usual, using an -omic variable as primary end-point. **(b)** Power of a Student’s t-test (two-tailed) as a function of effect size (Cohen’s d) and sample size (n = 3–6). Three alpha risk conditions are shown (0.05, 0.01, 0.001). The two dashed lines represent 80% and 90% power, respectively. It is clear that with reasonable effect size values (Cohen’s d ≤ 4), it is impossible to achieve 80% power with fewer than 6 subjects per group. The data were generated using GPower 3.1.

There is confusion between biological repeats, technical repeats, and repetition of the experiment (Landis et al., 2012; Vaux et al., 2012) (Figure 1A). The subject is important enough that the National Academies of Sciences, Engineering, and Medicine have published a 257-page book on the subject (National Academies of Sciences, Engineering, and Medicine, 2019).

The concept of duplicate and triplicate comes from the fact that when the mean of two or three repeats is taken, variability is divided by the square root of the number of repetitions. Thus, the error goes from 1 to 0.7 (n = 2) and then to 0.58 (n = 3). This is called the standard error of the mean. Because sampling from a single mouse allows to fill numerous wells on a plate, these repeats are very useful when dealing with ELISAs or cell cultures (where they increase the effect size and also allow outliers to be eliminated if needed, see, for example, Grubbs, 1969). These repeats are not so useful when dealing with laboratory animals or human volunteers. This is because there is confusion between biological and statistical meanings of the concept of error (as with that of sample). Two mistakes are also common. The first, called p-hacking, is frankly fraudulent: “I study five animals, I look, I repeat with five animals, I look, and I repeat the procedure until the difference is significant.” If we want to carry out an interim analysis, we must do so as in large phase 3 studies in humans. In order to avoid unnecessarily prolonging a study involving several hundred or thousand volunteers or patients, an interim review is conducted by an independent committee. The downside is that in this case, if the study is continued, the alpha risk must then be lowered because information has been obtained that partially lifts the blind: if the committee recommends continuing, it means that the molecule (or vaccine) does not have too many minor side effects and is potentially effective. This is the price to pay, and we usually go from 0.05 to 0.025.

The second problem relates with repetitions of the same experiment. When an experiment is repeated, the probability of obtaining a significant result in a second trial is not guaranteed (Killeen, 2005; Cumming, 2014). For example, two studies studying the same ‘effect’ (p < 0.05) would have a probability of both finding a significant result of 50% with a power of 0.5, a probability of 68% with a power of 0.8 and a probability of 81% with a power of 0.9. However, repetition is only conceivable in another laboratory, on the other side of the world, where it is hoped that the experimental biases will not be the same (Rotter et al., 2026). The point of this type of repetition is either to refute the initial hypothesis or to reinforce it through a regression to the mean effect (Held et al., 2020). Furthermore, how should the results be presented: as two studies, one of which may not be significant? By pooling the results? But pooling is highly questionable, and it would have been better to do an overall calculation from the outset. If the principle of repetition is linked to the fact that the experiments are long and that we want to split up the implementation, then this should have been included in the initial design and randomized, or better, distributed in a predetermined manner (there are randomization tables and software available for this). This would have minimized bias, particularly those related to time, learning curves, differences between multiple observers, etc. Furthermore, returning to the example given above of a two-way design, by increasing the number of animals per group from 10 to 20 (two experiments of 10), we can consider as a first approximation that we used an alpha risk of 0.002 instead of 0.05 for the calculation (with a Cohen’s f = 0.45 and a 80% power, see paragraph 4.4 and Box 2). Is it necessary to use so many animals when a rigorous experimental design and a calculation based on a more reasonable alpha risk of 0.01 or a power of 0.9 would have led to the use of only 15 animals per group?

Box 2 **ANOVA and regression.**

ANOVA and regression are two sides of the same model.

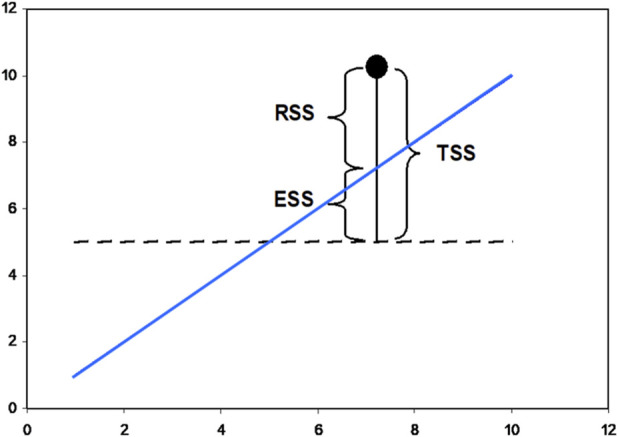

In regression and in ANOVA, the total sum of squares (TSS) is the sum of the explained (or between-groups) sum of squares (ESS) and of the residual (or within-groups) sum of square (RSS). Dividing these sums by their respective degree of freedom gives the corresponding mean squares. The ratio of the explained mean square over the residual mean square is the variance ratio (or Fisher-Snedecor statistics, F).

In statistics, a degree of freedom is equal to the number of observations or variables minus the number of relationships between those variables. For example, when comparing two means using a Student’s t-test, the number of df is equal to N - 2, where N is the total number of subjects. This is because knowing the mean of each sample allows us to know the value of the last subject of the sample if we know the others. For the numerator of the F statistics (explained mean square), it is the number of groups in the factor or interaction minus one.

In this text, for sake of simplicity and clarity, we only consider experimental factorial design with equal number of subjects and equal difference between means in each group.

The following table shows the summary of a two-way ANOVA (A*B) balanced (i.e., with equal number of subjects in each level of factors).

**Table:** 

Source of variation	df	Mean square	F
A	p-1	SSA/(p-1)	MSA/MSerr
B	q-1	SSB/(q-1)	MSB/MSerr
A*B (interaction)	(p-1)*(q-1)	SSAB/((p-1)*(q-1))	MSAB/MSerr
Residual	(n-1)*p*q	SSerr/((n-1)*p*q)	​

df is degree of freedom, p and q are the number of levels in groups A and B respectively, n is the number of replicates (observations) in each level in groups so that N = n*(p + q) and SSA, SSB, SSAB, and SSerr, are the sums of squares for factor A, factor B, interaction and error or residual respectively, MSA, MSB, MSAB, and MSerr, are the corresponding mean squares.

For a one-way ANOVA with groups of identical size and identical difference in means, Cohen’s f (effect size) is equal to half of Cohen’s d. Usually, because estimates of Cohen’s f are unavailable, we recommend the following approximations: for the main effects f = d/2; for the interactions f/2 (medium interaction) or f/4 (weak interaction). This may be considered conservative, but this is because we want to avoid insufficient *a posteriori* power (Gjerde, et al., 2004). Similarly, we recommend to take into account the comparison with the highest number of df. It is not very economical in terms of animals, but this ensures that the power of the final analysis will be kept high enough. For example, consider a two-way ANOVA (A and B) with 3 and 4 levels each. The main effects have 2 (3–1) and 3 (4–1) df. The interaction has 6 (2*3) df. It is clear that with this calculation based on the maximum number of df, the calculated number of subjects needed will be greater than if calculated considering the other (main) effects. We recommend also that the reader consult the G*Power3 manual pp 26–30.

### 2.6 Beyond experimental designs

Other approaches include sequential methods (Dixon’s up-and-down method or Bayesian methods used in phase 1 studies in humans, such as the Continual Reassessment Method [CRM]), which seek to identify a dose and/or effect with the lowest risk. These techniques are very effective in terms of reduction, but it is virtually impossible to calculate the exact number of animals required *a priori*, even using simulations. Only stopping criteria are available, along with an upper limit on the number of subjects. The *a priori* calculation is most often based on a heuristic that takes into account previous experiments and the literature.

Bayesian methods, which are very effective in terms of reduction are rarely used. However, when using the results of a previous study or addressing a project involving successive procedures, using a Bayesian method is very economical since previous results are usually incorporated into the final calculation. These methods are only just beginning to be used and will hopefully develop further in the future (Larson and Shockley, 2024; Blaß et al., 2025). The same principle (i.e., using previous data in a new experiment) integrates an inter-occasion variability factor into pharmacokinetic and pharmacodynamic models (see paragraph 4.6). Similarly, studies where the same individual is used multiple times during an experiment are preferable (longitudinal studies). In addition, the decrease in variance like in repeated measure ANOVAs add its effect.

## 3 Is it always necessary to reduce?

It is important to calculate the correct number of animals required and not to reduce the sample size excessively, as it would be more serious to have an insufficient number and have the article rejected due to lack of power for example. In that case, the animals used would have died for nothing. Also, it should not be forgotten that some experiments inherently involve exclusions or non-inclusions. For example, the implantation of tumors in mice, even immunodeficient ones, is often accompanied by a failure rate up to 20% or more. This must be taken into account and these animals must be added to the number obtained by the power analysis.

Two situations deserve to be highlighted: insufficient power of the test(s) and lack of correction for multiple comparisons.

### 3.1 Power of tests

Because the power of the tests depends on the number of animals, we calculated the power of a Student’s t-test (two-tailed) based on the number of subjects (with an identical number of subjects per group) and the effect size (Cohen d) using G*Power v. 3.1 software (Faul et al., 2007) (Figure 1B). It is clear that fewer than 6 subjects/group is totally insufficient to achieve 80% power. These results support those of many authors who warn against statistical mistakes that are so common in biology journals (Button et al., 2013; Ioannidis, 2005; Hayden, 2013; Nuzzo, 2014).

### 3.2 Correction for multiple comparisons (family-wise error rate [FWER] correction)

Imagine you are offered the chance to win $1 billion. To do so, you have to bungee jump with a 1 in 20 (0.05) chance that the bungee cord will break. You decide to take the risk. Would you agree to do it again once, twice, etc.,? Probably not, because each time the risk is still 5% and 1 day the bungee cord will break. We therefore need to apply what is known as a correction for multiple comparisons (Lee and Lee, 2018). When calculating the number of subjects required *a priori*, only the Bonferroni correction can be applied in a simple manner. This involves dividing the alpha risk by the number of comparisons to be made. If you have three groups, A, B, and C, and you make the three comparisons AB, AC, and BC, the alpha risk to be taken into account is 0.05/3, or 0.0167. This seems very complicated ! It is true, but since researchers always want to look at each tree and not the forest, they will want to compare group by group, even if there are dozens of them and some comparisons are of no interest. This is very penalizing and quickly leads to an unreasonable increase in the number of animals required. It is therefore essential to limit the number of planned comparisons in advance when calculating the number of animals required. However, adherence to this principle remains difficult unless, as in human studies, all study elements are pre-specified and recorded in a publicly accessible register (Zarin et al., 2016; EU, 2025). In addition, the construction of an appropriate experimental design is essential. To this end, ANOVAs with interaction allow the number of animals required to be limited.

The correction for multiple comparisons (FWER) should not be confused with the false discovery rate correction (FDR) (Benjamini and Hochberg, 1995), which is used in genomics where the number of comparisons quickly becomes extremely large. FDR (and further refinements of the procedure) has been conceived for large-scale testing and is exploratory rather than confirmatory (Figure 1A). However, -omics data comes from an animal, and it is the latter that must be considered for statistical analysis and *a priori* power analysis.

## 4 In practice, calculation of the number of subjects required, often referred to as power analysis

After designing the project and the experimental plan, the next step is to perform the power analysis, which allows the number of animals required to be calculated. We will not go into the details of the calculation itself, as software does this very well, provided that the experimenter has a good understanding of the process. We have considered that variables followed a normal distribution, which is certainly not always the case. However, it is difficult to perform a power analysis for non parametric (ditribution-free) tests, unless approximations are done.

### 4.1 Calculating the number of subjects required

**FIGURE 2:**
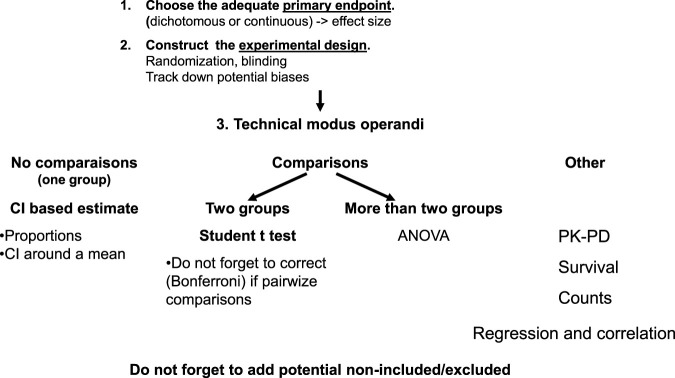
Planning the study and calculating the number of animals needed for each procedure.

This calculation requires choosing the alpha and beta risks (the power being equal to 1–beta). These risks must be reasonable, and choosing a low beta risk should not lead to an unjustified increase in the number of animals. A power of 95% is only justified in exceptional cases and should be thoroughly justified. Figure 2 represents the flowchart of decisions. A list of software is depicted in the appendix. In the following, we consider the modus operandi of calculation with G*Power3.1 as the default software.

### 4.2 Single cohort. calculation based on confidence interval

It may be desirable to measure a statistics drawn from a simple sample, for example, the proportion of mice exhibiting a certain trait in a new line, or the size of a tumor 30 days after implantation. A minimum degree of precision around the estimate is needed (margin of error) and then more than one animal should be tested. In this case, the effect size is a predetermined confidence level. If the variable is dichotomous (proportion), the Clopper-Pearson (so-called exact) method needs to be chosen. Be careful, since this method, which is not really exact, is very conservative and the number of animals calculated may be slightly less than needed. Some software is listed in the appendix for the calculation of CIs around a mean or a proportion. If the variable is continuous and approximately normally distributed, a predetermined confidence level around a coefficient of variation (CV) may also be considered: CV = SD/m, where SD is the standard deviation and m is the mean values, all these variables need to be estimated *a priori* as usual. Tables allowing for approximate calculations can be found in Kelley (2007). In addition, two R packages are listed in the appendix.

### 4.3 Comparison between two groups

#### 4.3.1 Continuous variables

To calculate the number of subjects required in a study where the endpoint is continuous (e.g., tumor size), it is necessary to know the expected difference and the variability of the measurement (Cohen’s d), and to determine alpha, beta (or power, 1-beta). When pairwise comparisons are planed, a correction for multiple comparisons is mandatory (see paragraph 3.2). Wilcoxon tests are distributions free. Asymptotic approximations exist and are implemented in the G*Power sofware.

#### 4.3.2 Proportions

This refers to the proportion of subjects with a particular characteristic, such as eye color. The proportion in the reference population must be known, and the expected proportion in the treated population must be estimated. For a comparison between two groups, Fisher’s exact test is the most appropriate. When there are more than two groups (such as eye color), the calculation is often done by considering pairwise comparisons with an adjusted alpha risk (see paragraph 3.2). The exact calculation involves the Chi2 distribution and Cohen’s w.

### 4.4 Comparison between more than two groups (analysis of variance, ANOVA)

These allow comparing more than two groups with each other. ANOVA is in itself a design of experiments (Box 2). ANOVAs offer the greatest savings when designing appropriate experimental designs, but power calculation may become complicated, involving the number of degrees of freedom (df), as well as the type of squares if the data are unbalanced (Faul et al., 2007). Since researchers usually do not have a statistician at their disposal, we will only consider groups of equal size in what follows. Interactions are the key to reduction, but this requires having a prior idea of the magnitude of these interactions. From a conservative perspective (i.e., not impacting the validity of the research), we will ensure that the principle of reduction and the choice of interactions size do not prevent the significance of the main effects (Gjerde, et al., 2004) (See G*Power3 tutorial pp 25–27). If we consider a Cohen’s f of 0.4 (roughly corresponding to a Cohen’s d of 0.8) for the main effects and interactions, 80% power, and an alpha risk of 0.05, the plan presented above (two groups A and B, each with two levels, 1 and 2) treated by a one-way ANOVA (4 groups) requires 76 animals, whereas if the interaction is taken into account in a two-way ANOVA, only 52 animals are needed. The saving is 32%.

ANOVAs for repeated measures (also called within-between) are frequently used, for example, in behavioral studies. This type of experimental design significantly reduces the number of animals required. Not only the same animals are used for several measurements, but the fact that the “within” data are correlated further reduces the variability. This is the advantage of longitudinal studies, where the same subject is used at several points of measure, which reduces variability.

### 4.5 Regression and correlation

In practice, there are three situations: 1) Is my regression slope significantly different from zero? 2) Do my groups have a slope and/or intercept that is significantly different from each other? 3) Are two correlation coefficients significantly different? For the first two situations, a t-test is used. To compare two correlation coefficients (Pearson’s r), Cohen’s q coefficient is used, which is the result of a Fisher transform that the G*Power software calculates automatically.

Examples: In an experiment testing the effect of an anticancer drug on Sprague-Dawley rats, we want to measure the effect of the drug compared to placebo on growth. From a previous experiment, we know that in the control animals, the weight-time curve is linear with a slope of 0.22 ± 0.07 g/day (mean ± standard deviation). We are looking to detect a difference of 0.1 g/day, i.e., an effect size of 1.43. Considering an alpha risk of 5% and a power of 80%, seven animals per group are necessary (one-tail t-test).

In another experiment, we want to test new antibodies for quantification of the colocalization of a marker on histology plates. The quality of the association is measured by the coefficients of Manders and Pearson. We know that Pearson’s r is usually equal to 0.61. We want to test the new antibodies on an independent control group, provided that the number of animals needed is not too large. Considering alpha and beta risks at 5% and 20% respectively, an expected Pearson’s r difference of 0.15, the effect size q, calculated by G*Power3.1 after a Fisher transform is 0.29. This requires 153 animals per group (unilateral z test). It appears that this number does not seem ethically reasonable.

### 4.6 Pharmacokinetic and pharmacodynamic studies

**FIGURE 3:**
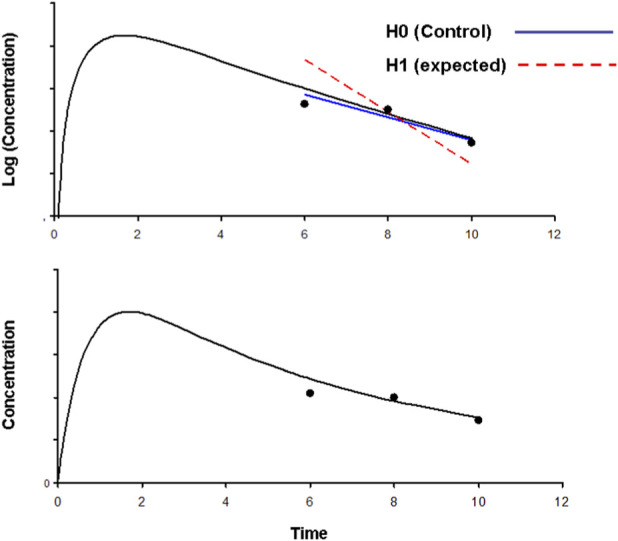
The principle of three points in the terminal phase. Bottom: Dose-effect curve after oral administration, with 3 points in the terminal (elimination) phase. Top: The same curve and points in a logarithmic representation. The terminal phase may be approximated by a single declining exponential. Then it is possible 1) to calculate the terminal time constant (τ) and the half-life (T1/2 = 0.693/τ) and 2) to extrapolate the total area under the curve (AU $C_{0‐\infty }$ ). The estimation of the number of animals needs only to compare the two slopes (absolute value of the difference between observed and expected slopes) by a Student’s t-test.

Pharmacokinetics is the study of absorption, disposition and elimination of foreign molecules (xenobiotics) in the body. It allows to summarize and condense information into a few derived parameters, such as half-life, clearance, and volumes. This section will not address Michaelis-Menten kinetics, but only its linear approximation at relatively low concentrations. Two approaches currently coexist regarding modeling: the so-called non-compartmental kinetics (which is considered semi parametric) and the so-called linear compartmental kinetics, which assumes that transfers between compartments are governed by constant rates. Compartmental kinetics is modeled by non-linear regression, except for the simple single-compartment model where a logarithmic transformation allows for linear regression analysis. Pharmacokinetic studies require the collection of samples, most often blood samples. In our case (principle of reduction), both approaches need that samples be taken sufficiently long after administration of the molecule to model the so-called terminal phase (Figure 3). It is assumed that three measurement points are necessary. The main problem in pharmacokinetic studies in animals is destructive sampling, where each animal is killed after a unique sample, as in many studies where a longitudinal model (or at least partly longitudinal model) might be particularly appropriate. This is why it is worth considering the possibility of using the same animal for more than one measurement point, ideally 2, 3 or more samples distributed evenly: for example, mouse A will be sampled at T0 and T3, mouse B at T1 and T4, mouse C at T2 and T5, etc. Similarly, combining two studies or incorporating the results of a pilot study might be of great value for reduction: It is simply a matter of adding to the parameter(s) an indicator (interoccasion variability parameter) linking the two occasions. This allows also to link two different conditions in the same individual. Kinetic studies are often analyzed using either inappropriate techniques (ANOVA) or obsolete methods. It is preferable to summarize the information using a few derived variables (half-life, clearance or area under the curve, etc.). In addition to non compartmental analysis, there are three parametric methods: in order, 1) Naive Pooled Data Analysis, which analyses the whole data set considering it as originating from a single average individual (rustic but fairly effective), 2) the Two-Stage method, which analyzes subject by subject but needs that each subject be sampled throughout the observation time and 3) analysis by nonlinear mixed-effects modeling (Patron-Bizet et al., 1998; Ette and Williams, 2004). When each animal is sampled only once (destructive sampling), only the naïve pooled data analysis is possible, for both the non compartmental and compartmental analysis. The two-stage method is rarely possible because it needs that the same subject be sampled at all time-points. In terms of reduction, the mixed-effect (population) modeling technique is by far the best method (with the restriction that most animals must have more than one or two point(s) of measure) In addition, most specialized software such as NONMEM include a simulation module that allows the number of animals required to be calculated easily (see the appendix for a list of the few software allowing mixed-effect modeling).

Some guidelines can be given for calculating the number of animals required, bearing in mind that pharmacokinetic parameters are log-normally distributed (Figure 3). As with all the procedures described above, one parameter must be chosen (primary endpoint) and the calculation performed assuming that this parameter represents the behavior of the others. It is always necessary that sampling covers the terminal (elimination) phase. If the choice of mixed effect modeling is made, two samples must be simulated, the first (control) with the known parameters from the literature, and the second with the expected effect of the treatment on the chosen parameters (most often clearance or terminal half-life).

#### 4.6.1 Pharmacodynamics and dose-response curves

First, it is important to differentiate between continuous and binary data. Continuous effects follow Hill’s law, while 0/1 effects are probabilistic (the higher the dose, the greater the probability of the effect occurring or *vice versa*). They follow a logistic law. All these sigmoid functions are generally treated in the same way as kinetic studies and once again, mixed-effect modeling represents the better choice. However, authors are often looking for a dose (ED50, IC50, LD50, P50, etc.) or simply seeking to compare two or more groups. This is why the principle of linearization is simple and perfectly suited, at least for calculating the number of subjects required. For continuous effects, the log (Dose)-Effect relationship can be approximated by a straight line in the 25%–75% effect range. All-or-nothing effects are often treated by a logit or probit transform for the same reasons. Thus, it is sufficient to choose an interval in advance for the search for a single dose to be used in further procedures (pilot study), or to treat the comparison of several groups as a comparison of straight lines (see the regression section, paragraph 4.5). If possible, using the same animal for several points greatly reduces variability (longitudinal analysis).

Dose escalation and sequential techniques which are used for phase 1 studies in humans allow to estimate ED50, IC50, LD50 with very few subjects. They are very specific and readers are referred to specialized literature (O'Quigley et al., 1990; Oron et al., 2022; Fils et al., 2023). These techniques do not allow calculating the number of subjects needed for a specific study and only either a bibliography based heuristic approach (up-and-down techniques) or stopping rules (Continuous reassessment method, CRM) are possible.

### 4.7 Survival analysis

Survival analysis studies the time span between an individual’s entry into a study and the occurrence of an event. A distinctive feature of methods studying survival is the consideration of censored observations, which rarely occurs in our animal facilities (it is not common for a mouse to escape from its cage). There are usually two types of approaches: the survival function, typically represented by the Kaplan-Meyer graph, and the hazard function (semi-parametric Cox model). The latter model is very powerful but requires the intervention of a statistician. Usually biologists use the Kaplan-Meyer graph combined with the log rank test (sometimes called Mantel-Cox test) for comparing curves. When comparing multiple curves pairwise, a correction for multiple comparisons (Bonferroni correction) must be applied. Similarly, if the curves include fewer than 6-8 subjects, a continuity correction is needed. Several software allows the calculation of sample sizes, often on-line. Some of these software are listed in the appendix. However, in the case of survival analysis, the inherent problem with the power analysis is that the effect size is the number of events, which is not always easy to estimate a-priori.

Similar to the Cox model, the occurrence of an event can be modeled parametrically. Because there is no censoring, the model can be very simple. An example is a decreasing exponential, which can be analyzed by linear regression after logarithmic transformation (see paragraph 4.4). More complex models can be used by using the simulation capabilities of pharmacokinetic software, some of which is free (Moine and Mazoit, 1999).

### 4.8 Count data (Poisson distribution and process)

The Poisson distribution applies to counting events such as births, ovulation, mutations, etc. The events must be relatively rare, otherwise the distribution will be closer to a normal distribution. This type of calculation requires the assistance of a statistician who can assess the relevance of the model and perform the power calculation.

## 5 Conclusion

In conclusion, at a time when societal pressure and the methodological rigor demanded by the editors of medical and biology journals are becoming more pressing, it is important that all stakeholders participate. Biology still lags far behind human medicine in terms of blind procedures, randomization, and calculating the number of subjects required. *A priori* calculation of the number of subjects required has become essential. Good practice recommendations organizations such as ARRIVE and the EQIPD framework consider that planning statistical analysis before conducting the experiment is of major importance (ARRIVE Guidelines, 2025; Vollert et al., 2022). However, these recommendations are too vague and provide a framework rather than a practical guide. It is why this article aims to give a practical framework for determining the appropriate number of animals required in experimental biology and pharmacology. In addition, we may recommend that editors of biology journals require authors to have registered their project on a registry such as PreclinicalTrial.eu (2025) or Animalstudyregistry.org (2025) before the start of the trial as is done in humans.

## Footnotes

1 Here, m1 and m2 are the mean values in groups 1 and 2, and SD is the overall standard deviation. In the case of power calculation, SD is considered to be the standard deviation of the reference population.

## Appendix A

This appendix lists a series of the most common software and utilities.

### Protocol design and experimental plans

Our British colleagues’ website is extremely helpful, particularly its graphical interface: https://eda.nc3rs.org.uk/.

For randomization

https://www.randomizer.org/ and https://ctrandomization.cancer.gov/.

Animal studies registries; https://preclinicaltrials.eu/, https://www.animalstudyregistry.org/asr_web/.

### Power analysis

The best general software is G*Power v 3.1. It is free and very comprehensive:

https://www.psychologie.hhu.de/arbeitsgruppen/allgemeine-psychologie-und-arbeitspsychologie/gpower.

It comes with a fairly simple tutorial and a manual that requires a minimum of statistical knowledge.

### Confidence interval of proportions

https://sample-size.net/confidence-interval-proportion/.

A simple Excel sheet is available upon request from an author (JXM).

For coefficient of variation at least two procedures are available in R: sscv from the package BE and the package MBESS.

### ANOVAs

Either G*Power or for those who use **R** (https://cran.r-project.org/), the easypower procedure is recommended. It is easy to use and handles all interaction. You just need to choose the appropriate f values for the main effects and interactions, whereas in G*Power, only a single f value is possible, thus necessitating to perform a multiple step procedure. For all software, the only limitation is that the design must be balanced with groups of identical size.

ANOVAs for repeated measures. As with factorial ANOVAs with multiple factors and interactions, it is not always easy to choose Cohen’s f. In G*Power, simply choose F tests, ANOVA Repeated measures, within-between interaction. In R, the wp. rmanova procedure from the WebPower package also makes it easy to calculate the number of subjects required.

### Survival analyses

Sample size: https://sample-size.net/sample-size-survival-analysis/.

MedCalc Software Ltd. Sample size for Survival analysis (log rank test). https://www.medcalc.org/en/calc/sample-size-survival-analysis.php (Version 23.4.9; accessed 27 February 2026).

PS Power and Sample Size from Vanderbilt University, survival tab. https://biostat.app.vumc.org/wiki/Main/PowerSampleSize.

### Procedures in R

There are also numerous procedures in R. To find them, go to the CRAN website and search using the keyword “power” https://cran.r-project.org/.

For example, pwr https://cran.r-project.org/web/packages/pwr/index.html and TrialSize https://cran.r-project.org/web/packages/TrialSize/.

### Pharmacokinetics

Pharmacokinetics and pharmacodynamics. Mixed-effect (population) modeling: NONMEM, Phoenix NLME, Monolix, the latter free, are software especially devoted to PK-PD modeling with the possibility of simulation (Monte-Carlo type). The are also procedures in R.
